# Pathological Research in London County Asylums

**Published:** 1904-11-05

**Authors:** 


### PATHOLOGICAL RESEARCH IN LONDON COUNTY ASYLUMS.

Much credit is due to the Asylums Committee of the London County Council for the measures they have adopted from time to time with the purpose of improving the general conditions and welfare of the patients in the great asylums which are under their supervision. There is one innovation which they introduced a few years ago, namely, the institution of a research laboratory under the control of an expert pathologist, which, perhaps, more than all others, is likely to have beneficial and far-reaching results. Asylum medical officers have but little leisure for pathological research; their time is fully occupied with clinical and administrative duties. Moreover, in many respects a special training and a special disposition are necessary attributes of a skilled pathologist, and the appointment of such an individual, whose whole time would be devoted to research into the varying pathological conditions affecting the insane, held from the first the promise of much fruitful achievement. And it will hardly be denied that the promise has already been amply fulfilled by the results of inquiries which have been reported periodically by Dr. Mott. Early in the year we called attention to a report by Dr. Eyre, bacteriologist to Guy's Hospital, who, on the recommendation of Dr. Mott, carried out investigations with regard to the bacteriology of certain intestinal maladies, as a result of which he was able to report the discovery, in a number of cases of asylum dysentery, of a bacillus undistinguishable from Shiga's B. dysenteriæ; and although the number of cases examined was too small to permit of general conclusions, yet the results strongly suggested that Shiga's bacillus is the cause of asylum dysentery; an observation which, if ultimately verified, will not only establish an important scientific fact, but will afford a basis for rational preventive measures which will probably lead at no very distant date to the abolition of this scourge from our asylums.

Another interesting paper is contained in the recently published Annual Report of the Asylums Committee in the form of some preliminary observations by Dr. Mott on the occurrence of tuberculosis in the insane. It is a recognised fact that this disease is largely prevalent in asylums, and, apparently, in some asylums more than others. With a view of obtaining accurate data for comparison of the percentage of tubercle acquired in an old asylum with a new and modern one, Dr. Mott undertook to make all the post-mortem examinations at Colney Hatch for a period of one year, in order that the results might be compared with those obtained at Claybury. As the twelve months were not completed at the time of his report to the Asylums Committee Dr. Mott refrains from giving any statistics, but nevertheless he expresses the opinion that post-mortem appearances indicate that, in a majority of cases of tubercle, the disease is not acquired in the asylum. Such a statement, provisional though it be, is very welcome, and whatever truth there may ultimately prove to be in the proposition, it is in the meantime satisfactory to know that an attempt is in active progress to solve the problem of tuberculosis among the insane.

As a point of interest it may be mentioned that Professor Koch of the University of Missouri has carried out in the laboratory at Claybury some valuable investigations on the chemistry of the brain, and Dr. Gordon Wilson, another prominent American, has been engaged in a research relating to the structure of the peripheral nerve endings in tissues by a special method which he has introduced. So that the laboratory has been of direct service to others than our own countrymen. It is greatly to be hoped that the pathological laboratory at Claybury will prove to be the means of stimulating the profession to take a livelier interest in neurological research, and that medical men in general as well as asylum officers will have a keen regard to the reports which emanate from Dr. Mott and his assistants.

